# Integrative multi-omics analysis prioritizes ALDH2 as a candidate Puerarin-associated target linked to macrophage infiltration in lung adenocarcinoma

**DOI:** 10.3389/fimmu.2026.1918481

**Published:** 2026-09-16

**Authors:** Yufeng Chen, Zhujuan Zhong, Guoyuan Ju, Yuanyuan Cai, Lina Zhou, Qianyu Wang, Bo Yang

**Affiliations:** 1 Department of Pathology, Jiangsu Province (Suqian) Hospital, Suqian, Jiangsu, China; 2 The Quzhou Affiliated Hospital of Wenzhou Medical University, Quzhou People’s Hospital, Quzhou, Zhejiang, China; 3 Department of Oncology, The Affiliated Suqian Hospital of Nanjing University of Chinese Medicine, Suqian Hospital of Traditional Chinese Medicine, Suqian, Jiangsu, China

**Keywords:** ALDH2, immune microenvironment, LUAD, macrophage, network pharmacology, NSCLC, Puerarin

## Abstract

**Background:**

Puerarin is a bioactive isoflavone with reported anti-tumor and immunomodulatory activities; however, its candidate molecular targets and associations with the immune microenvironment in lung adenocarcinoma(LUAD) remain incompletely defined. This study integrated computational analyses with a murine LUAD model to prioritize candidate puerarin-associated targets and evaluate their relationships with immune-cell changes.

**Methods:**

We first identified puerarin targets, non-small cell lung cancer (NSCLC)-associated genes, and differentially expressed genes (DEGs). An integrative strategy combining network pharmacology, multi-omics, and machine learning algorithms (Least Absolute Shrinkage and Selection Operator, Support Vector Machine-Recursive Feature Elimination, and Random Forest) was employed to screen feature genes. Feature genes were further evaluated by molecular docking, molecular dynamics simulations, Mendelian randomization(MR), cellular origins and immune infiltration analyses, and *in vivo* animal experiments using an orthotopic LUAD xenograft model.

**Results:**

A comprehensive analysis identified 130 targets associated with puerarin, 10,871 genes related to NSCLC, and 4,690 DEGs, resulting in an intersection comprising 39 candidate genes. Five feature genes (ABCG2, ALDH2, MIF, PTGS1, and SELP) were retained by three machine-learning approaches and ABCG2, ALDH2, MIF, and SELP showed consistent diagnostic performance in the training and validation cohorts. Docking and molecular dynamics simulations predicted favorable and stable modeled interactions of puerarin with ABCG2 and ALDH2, but did not establish direct target engagement. MR using a LUAD outcome dataset suggested an inverse association between genetically predicted ALDH2 expression and LUAD risk. Single-cell analysis indicated relative enrichment of ALDH2 transcripts in macrophages. *In vivo*, puerarin reduced orthotopic tumor burden and was accompanied by increased total ALDH2 expression and higher proportions of F4/80^+^CD11b^+^ cells in peripheral blood and tumor tissue.

**Conclusion:**

Puerarin suppressed tumor growth in a murine LUAD model and was associated with ALDH2 upregulation and increased macrophage infiltration. Integrated analyses prioritize ALDH2 as a candidate molecule potentially linking puerarin treatment to changes in the tumor immune microenvironment. Direct puerarin–ALDH2 binding, macrophage-specific ALDH2 regulation, and the functional consequences for macrophage polarization require further experimental validation.

## 1 Introduction

Lung cancer is among the most common malignant tumors and is the leading cause of cancer-related deaths worldwide. The International Agency for Research on Cancer estimates that in 2022, lung cancer was the most commonly diagnosed cancer globally, accounting for approximately 2.5 million new cases, or 12.4% of all cancer diagnoses (1). Additionally, lung cancer was responsible for approximately 1.8 million deaths, representing 18.7% of all cancer-related fatalities. Non-small cell lung cancer (NSCLC), the predominant form of lung cancer, is associated with high morbidity and mortality rates, with lung adenocarcinoma (LUAD) being the most common histological subtype (2). Despite ongoing advancements in diagnostic and therapeutic strategies, the survival rates for patients with lung cancer remain suboptimal (3). Therefore, exploring new treatment paradigms is urgently required.

Traditional Chinese medicine (TCM), known for its historical depth, clinical expertise, holistic view, and personalized treatments, has attracted growing interest for its potential function in the prevention and control of lung cancer (4). Puerarin, a monomer found in TCM, is a bioactive isoflavone extracted from the roots of Kudzu and the Pueraria genus (5). Extensive research has recently been conducted on puerarin, encompassing both scientific and clinical studies. Since puerarin has demonstrated several beneficial effects in various diseases, including metabolic dysfunction-associated fatty liver disease (6), diabetes mellitus and its complications (7, 8), cardiovascular diseases (9), cerebrovascular diseases and Parkinson’s disease (10), ulcerative colitis (11), osteoarthritis (12), cancers (13, 14), and others, it has been attracting increasingly intense attention worldwide. Recent studies suggest that puerarin can reduce pulmonary fibrosis (15), improve acute lung injury (16), and hinder the development of pulmonary hypertension induced by hypoxia or monocrotaline (17), although research on its effects on lung cancer remains limited.

Network pharmacology (NP) is an emerging discipline grounded in systems biology, focusing on the analysis of biological system networks and the design of multi-target drug molecules through specific signal node selection (18). NP is a method for analyzing and visualizing correlations among drug components, targets, and diseases. NP provides a novel methodological approach to comprehending traditional medicine holistically, leading to advancements such as TCM-NP. Advancements in artificial intelligence (AI) necessitate the development of network-based AI methods in NP to elucidate treatment mechanisms of complex diseases using extensive omics data (19).

Molecular docking has become an essential part of driving drug discovery and biological research (20). Docking is most frequently used to predict interactions between protein targets and small molecules, which can predict the bound structure and binding energy of a compound. Docking predicts target-ligand interactions and various ligand conformations at different positions to identify novel therapeutic molecules. This prediction indicates the effectiveness of a molecule or its varying affinity for the target (21). Molecular dynamics (MD) simulations were performed on the ligand-receptor complexes to evaluate the binding stability and conformational adaptability between active compounds and therapeutic targets.

Although ALDH2 has been implicated in tumor progression and immune regulation, its relevance as a therapeutic target in LUAD and its potential interaction with puerarin have not been systematically investigated. In this study, we adopted a multi-pronged integrative approach—incorporating NP, machine learning, molecular docking, MD simulations, and Mendelian randomization (MR)—to screen for puerarin targets in LUAD, with a specific focus on ALDH2. Furthermore, single-cell data suggested relative enrichment of ALDH2 transcripts in macrophages. *In vivo* experiments were further performed to assess puerarin’s therapeutic efficacy and its possible effects on the tumor immune microenvironment. Our findings suggest ALDH2 to be a candidate mediator worthy of further exploration, though the current data do not establish direct target engagement or definitive causal relationships.

## 2 Materials and methods

### 2.1 Data source and preprocessing

#### 2.1.1 Transcriptome data

The NSCLC training dataset, GSE19188, used in this study was sourced from the Gene Expression Omnibus (GEO, https://www.ncbi.nlm.nih.gov/geo/) database (22). GSE19188 consisted of 156 lung tissue samples, including 65 adjacent normal lung tissue samples and 91 NSCLC tissue samples. The NSCLC dataset, GSE268175, was retrieved and used as the validation set for this study. This dataset comprises 67 lung tissue samples, including 33 adjacent non-cancerous tissues serving as the control group and 34 NSCLC tissue samples constituting the case group. The training dataset used the GPL570 Affymetrix Human Genome U133 Plus 2.0 Array for microarray analysis, while the validation dataset was derived from the GPL16791 Illumina HiSeq 2500 through high-throughput sequencing.

Derived from two patients with NSCLC, the single-cell RNA sequencing dataset GSE198099 comprised two tumor tissues and their paired adjacent normal lung tissues. The experiment type was high-throughput sequencing, and the platform was GPL24676 Illumina NovaSeq 6000.

#### 2.1.2 The data for MR

Whole-blood expression quantitative trait locus (eQTL) summary statistics for the four feature genes were obtained from the eQTLGen batch in the IEU OpenGWAS Project. The OpenGWAS exposure identifiers were eqtl-a-ENSG00000118777 (ABCG2), eqtl-a-ENSG00000111275 (ALDH2), eqtl-a-ENSG00000240972 (MIF), and eqtl-a-ENSG00000174175 (SELP). The eQTLGen dataset was released in 2019 and included up to 31,684 participants of predominantly European ancestry. The outcome dataset was finn-b-C3_NSCLC_ADENO from the FinnGen collection in IEU OpenGWAS. This phenotype represents non-small cell lung cancer, adenocarcinoma, rather than all NSCLC. The outcome dataset contained 218,792 participants (571 cases and 218,221 controls), 16,380,466 single nucleotide polymorphisms (SNPs), and participants of European ancestry; the OpenGWAS data release used in this study was reported in 2021. Exact participant recruitment years were not available in the OpenGWAS metadata. Because the outcome case number was limited and both exposure and outcome data were predominantly European, the MR analysis was interpreted as supportive evidence for target prioritization rather than definitive causal proof or evidence generalizable to all ancestral populations.

#### 2.1.3 Puerarin targets

Puerarin’s Canonical SMILES and chemical structure were retrieved from PubChem (https://pubchem.ncbi.nlm.nih.gov/). Puerarin targets were identified using the Similarity Ensemble Approach (https://sea.bkslab.org/), Swiss Target Prediction (http://www.swisstargetprediction.ch/) and the STITCH database (http://stitch-db.org/). Duplicate data were eliminated after the merge.

#### 2.1.4 NSCLC targets

The GeneCards (https://www.genecards.org/) and Comparative Toxicogenomics Database (CTD) (https://ctdbase.org/) databases were used to identify potential targets associated with NSCLC.

### 2.2 Differentially expressed genes analysis

The limma software package (23), specifically version 3.54.2 (https://bioconductor.org/packages/2.7/bioc/html/limma.html), was used to identify differentially expressed genes (DEGs) with significant expression differences between NSCLC and normal control samples. The log_2_FC absolute value was established to be greater than 0.5, with a corrected *p*-value of less than 0.05 (24). DEGs in the chip data were visualized using a volcano plot (ggplot2 software package, version 3.5.1, https://github.com/tidyverse/ggplot2) and a heatmap (Complex Heatmap software package, version 2.14.0, https://bioconductor.org/packages/release/bioc/html/ComplexHeatmap.html). Puerarin target genes, NSCLC-associated genes, and DEGs were intersected using Venn to produce a Venn diagram. Intersecting genes were identified as candidate targets.

### 2.3 Gene ontology and Kyoto encyclopedia of genes and genomes enrichment analyses

To further explore the potential biological roles and signaling pathways of the candidate targets, Gene Ontology (GO) and Kyoto Encyclopedia of Genes and Genomes (KEGG) enrichment analyses were conducted on the intersecting genes using the “clusterProfiler” package (version 4.6.2, https://bioconductor.org/packages/release/bioc/html/clusterProfiler.htm) in R software, with a significance threshold of *p* < 0.05.

### 2.4 Construction of protein-protein interaction networks

Protein-protein interaction (PPI) networks for the overlapping genes were constructed using the STRING database (https://string-db.org/). Interactions with a combined score > 0.4 were considered significant. Cytoscape software was used to visualize the discovered PPI networks.

### 2.5 Screening of feature targets via three machine learning algorithms

Using the identified candidate targets, three robust machine learning algorithms were applied to highlight feature targets in NSCLC. To identify the most critical genes associated with the signature, three methods were applied: Least Absolute Shrinkage and Selection Operator (LASSO) regression, random forest (RF) algorithm, and Support Vector Machine-Recursive Feature Elimination (SVM-RFE).

Lasso regression, introduced by Robert Tibshirani in 1996, is an innovative method for variable selection. We used the intersecting genes to construct a machine learning algorithm, with the input dataset being the training set. Dimensionality reduction was achieved using the glmnet package (version 4.1.8; https://cran.r-project.org/web/packages/glmnetr/index.html) in R, configured with the family parameter as “binomial” to ensure analytical robustness. This allowed us to generate the Lasso coefficient path plot and the mean squared error path from Lasso cross-validation.

RF is a type of ensemble classification model. The “random Forest” package (version 4.7.1.2, https://rpkg.net/package/randomForest) was used to conduct RF analysis on the training set. Using the RF algorithm, the intersecting genes were ranked based on their importance scores, specifically the “mean decrease accuracy” and “mean decrease Gini” values.

Feature selection was performed using SVM-RFE, and ten-fold cross-validation identified the standard genes. Support vector machine analysis was conducted using the “e1071” package (version 1.7.13, https://CRAN.R-project.org/package=e1071). Here, the input dataset was the training set, and a radial basis function kernel was independently tuned for the datasets of ten-fold cross-validation (CV). This involved an internal ten-fold cross-validation error estimation using a grid search for each combination of SVM hyperparameters. The final subset of features minimized the generalization error while maintaining predictive performance.

The genes that were simultaneously selected by all three algorithms were defined as the feature genes for subsequent analyses.

### 2.6 Identification of the key targets

The identified feature genes were incorporated into the training and test sets for expression level analysis. Targets with significant expression levels and consistent expression trends in both datasets were considered candidate key targets. Moreover, the area under the curve (AUC) was used to evaluate diagnostic accuracy. Receiver operating characteristic (ROC) curves for the potential key targets in the training and test sets were plotted using the “pROC” package (version 1.18.4; https://CRAN.R-project.org/package=pROC). Candidate targets with an AUC > 0.7 were selected as the key targets in this study.

### 2.7 Nomogram model construction

A nomogram is a graphical tool used to illustrate the relationships between multiple variables. A nomogram was developed in the training set using the R package “rms” (version 6.7.1; https://CRAN.R-project.org/package=rms) to elucidate the relationship between each key target and disease occurrence. The total score was calculated by summing the values of each factor on the nomogram. The total score can predict disease likelihood, with higher scores indicating an increased risk.

Subsequently, the calibration curve was plotted using the calibrate function from the R package “rms.” The calibration curve visually represents the results of the Hosmer–Lemeshow goodness-of-fit test outcomes. The model exhibits improved calibration performance when the predicted rate closely matches the actual incidence rate and the Hosmer–Lemeshow test produces a *p*-value exceeding 0.05.

We used the decision_curve function from the R package “rmda” (version 1.6; https://CRAN.R-project.org/package=rmda) to generate decision curve analysis (DCA) curves and assess the clinical utility of our predictive model. After an intervention was applied to the model, the decision curve of the model remained above the reference lines for “treat all” and “treat none” across most or all threshold probability ranges. This suggests the model could be valuable in various clinical applications. Furthermore, if the net benefit curve of the model maintains a high net benefit across several threshold probabilities and reveals stable variation, it may suggest that the model has strong generalization ability and consistent performance.

### 2.8 Gene set enrichment analysis of key targets

Spearman correlation analyses were conducted using the R package “psych” to examine the biological functions and pathways associated with key targets by calculating correlation coefficients between each target and all other genes in the training set. The coefficients were ordered from highest to lowest, and the R package “clusterProfiler” was used to perform gene set enrichment analysis (GSEA) on these ranked gene lists. Pathways with an adjusted *p*-value < 0.05 were deemed significant, and the top five were selected for visualization and further analysis.

### 2.9 Molecular docking

Molecular docking analysis was conducted via the CB-DOCK2 online server (https://cadd.labshare.cn/cb-dock2/php/index.php) to evaluate the binding potential between puerarin and identified key targets. The three-dimensional (3D) crystal structures of the essential target proteins were sourced from the RCSB Protein Data Bank (PDB) and downloaded as PDB files for subsequent molecular docking analyses. The 3D structure of puerarin was obtained from PubChem (https://pubchem.ncbi.nlm.nih.gov/) in the Structure-Data File format for molecular docking studies. Molecular docking of key target proteins with puerarin was conducted using the CB-DOCK2, with the binding free energy (ΔG, kcal/mol) serving as the main measure of interaction affinity. Molecular docking results were visualized using PyMOL and Discovery Studio to characterize predicted ligand–protein interaction modes.

### 2.10 MD simulations

GROMACS 2024.2 was used to perform MD simulations of the docked protein–puerarin complexes to evaluate the stability of the modeled interactions under the applied simulation conditions. The protein was modeled using the AMBER14SB force field parameters, while the ligand employed the GAFF parameters. The TIP3P explicit water model with periodic boundary conditions was selected. MD simulations over 100 ns verified the stability of the docked complexes while maintaining a constant temperature and pressure of 300 K and 1 bar, respectively, throughout the equilibrium phase.

Human-related protein sequences were extracted from the NCBI database (https://www.ncbi.nlm.nih.gov/). The AMBER GAFF force field was constructed using the Sobtop program (Version 1.0 dev5, http://sobereva.com/soft/Sobtop). Topology and parameter files for the protein and small-molecule ligands were generated using GROMACS (version 2024.2), employing AMBER14SB for the protein and GAFF for puerarin. The simulation box dimensions were defined, and periodic boundary conditions were applied, after which they were solvated with water molecules. To maintain system electroneutrality, some solvent molecules were substituted with Na^+^ and Cl^–^ ions, achieving a concentration of 0.15 mol/L. The system underwent sequential equilibration, first in an NVT ensemble at 300 K for 100 ps to stabilize the temperature, followed by an NPT ensemble at 1 bar for 100 ps to equilibrate the pressure. The simulation results were analyzed and visualized using DuIvyTools (https://github.com/CharlesHahn/DuIvyTools/tree/v0.6.0).

### 2.11 MR analysis

The extract_instruments function was used to obtain and filter instrumental variables by selecting SNPs strongly associated with exposure with a threshold of *p* < 1 × 10^–5^. To mitigate potential biases from linkage disequilibrium (LD), SNPs exhibiting LD were excluded using the ieugwasr package in R (version 0.2.1, https://github.com/MRCIEU/ieugwasr) with parameters r^2^ = 0.1 and kb = 1000.

Using the GWAS summary data for LUAD as the outcome and previously identified instrumental variables (IVs), we excluded IVs significantly associated with the outcome to minimize potential pleiotropy. We used the TwoSampleMR package (version 0.6.4, https://github.com/MRCIEU/TwoSampleMR) in R to align effect alleles and sizes between exposure and outcome datasets. Finally, we conducted MR analysis by aligning the exposure factors, IVs, and outcome data.

We retained only SNPs with minor allele frequency (MAF) > 1% to exclude rare variants with unstable effect estimates. SNPs with an F-statistic > 10 were identified as valid IVs, adhering to the conventional threshold for strong instruments.

We used five different MR methods—MR Egger, Weighted median, Inverse variance weighted (IVW), Simple mode, and Weighted mode—to investigate the causal relationships between key targets and LUAD. For multiple exposure factors analyzed separately, we selected candidate exposure factors with IVW-derived *p*-values < 0.05 as potentially relevant for subsequent analysis, with the results primarily based on the IVW method.

We assessed heterogeneity using the mr_heterogeneity function. When notable heterogeneity was observed (*p* < 0.05), the random-effects IVW model was applied; otherwise, the fixed-effects IVW model was used.

We evaluated potential horizontal pleiotropy using the mr_pleiotropy_test and run_mr_presso functions. A pleiotropy test *p*-value (pleio_P) > 0.05 suggested no significant horizontal pleiotropy, confirming the results’ reliability. Additionally, a heterogeneity Q-test *p*-value (het_qvalue) > 0.05 indicated no substantial heterogeneity in the analysis.

### 2.12 Transcriptome-based assessment of immune cell infiltration

Given the important role of immune cells in NSCLC development, we further assessed immune infiltration levels.

First, the “ssGSEA” algorithm from the “GSVA” package (version 1.46.0, https://www.bioconductor.org/packages/release/bioc/html/GSVA.html) was applied to quantify the relative enrichment levels of 28 immune cell types in both NSCLC patients and normal controls within the training set. This analysis generated infiltration scores, indicating the abundance of each immune cell type in each sample. The results were then visualized using stacked bar plots created with the R package “ggplot2.”

We used the “corrplot” package (version 0.92, https://CRAN.R-project.org/package=corrplot) to perform correlation analysis and visualize the differentially abundant immune cells. Correlations between differential immune cells and key molecular targets were evaluated using the “psych” package (version 2.3.9, https://CRAN.R-project.org/package=psych), and the results were visualized with “ggplot2”.

### 2.13 Single-cell RNA sequencing analysis

We used the Seurat package (version 5.0.1, https://CRAN.R-project.org/package=Seurat) for scRNA-seq data analysis and ensured that all samples were annotated. The following quality control steps were implemented: Retained cells expressing ≥ 200 genes and ≤ 4,000 genes to exclude low-quality cells and potential doublets. Cells with > 20% mitochondrial gene expression (indicating compromised cell viability) were removed. Genes detected in < 3 cells were excluded to ensure robust expression signals, and cells with total UMI counts > 20,000 (potential doublets or artifacts) were removed.

For principal component analysis (PCA), we selected the 2,000 genes with the greatest expression variability. Subsequently, both t-distributed stochastic neighbor embedding (t-SNE) and Uniform Manifold Approximation and Projection (UMAP) were applied for dimensionality reduction and visualization using the first 21 principal components (PCs) obtained from the PCA.

We performed cell clustering and sub-clustering analyses using the FindNeighbors and FindClusters functions from the Seurat package, applying an optimized resolution parameter of 0.40 to identify biologically relevant clusters. UMAP was used to intuitively visualize the identified cell clusters and sub-clusters.

The “FindAllMarkers” function with parameters “min.pct = 0.6”, “only.pos = TRUE”, and “logfc.threshold = 0.5” was applied to detect positive marker genes among different groups. Differential expression analysis was performed using the Wilcoxon rank-sum test. To determine cell subgroup types, we compared their marker genes with those of each cell type in the CellMarker database (http://xteam.xbio.top/CellMarker/). We then examined the expression of the main target genes in various cell populations.

### 2.14 TCGA-based evaluation of ALDH2 expression in NSCLC

We evaluated ALDH2 mRNA expression levels using The Cancer Genome Atlas (TCGA) (https://portal.gdc.cancer.gov/) data by comparing LUAD and lung squamous cell carcinoma (LUSC) tumor tissues with adjacent normal tissues.

### 2.15 Survival analysis of ALDH2 expression in LUAD

The prognostic significance of ALDH2 in LUAD was assessed through survival analysis using TCGA data. Patients were categorized into high- and low-expression groups according to the median ALDH2 expression level. The Kaplan–Meier method with log-rank tests, using the survival (version 3.5-5) and survminer (version 0.4.9) R packages, was employed to compare overall survival (OS) differences between the two groups. Effect sizes were estimated as hazard ratios with 95% confidence intervals; two-tailed *p* < 0.05 was considered statistically significant.

### 2.16 Animal experiments and drug therapy

Male C57BL/6 mice (4–5 weeks old) were purchased from Hangzhou Ziyuan Laboratory Animal Technology Co., Ltd. (Hangzhou, China). Before modeling, the mice were housed at 25 ± 1 °C and 50% relative humidity under a 12-h light/dark cycle and received a standard laboratory diet for 4 weeks. An orthotopic LUAD model was established in 8–9-week-old mice (20–22 g) using 2 × 10^5 LLC-luc cells. Mice were anesthetized via inhalation of isoflurane (RWD Life Science, China). Induction was carried out with 4% isoflurane, followed by maintenance at 2% isoflurane in 100% oxygen (flow rate: 1 L/min). Twelve tumor-bearing mice were randomly allocated in a 1:1 ratio to the vehicle-control group or the puerarin-treatment group (n = 6 per group). Puerarin was administered intraperitoneally at 50 mg/kg every 2 days for 2 weeks, beginning the day after tumor inoculation. The selected dose falls within a commonly used and tolerated range in previous murine studies of puerarin; however, no dose-ranging experiment was conducted in the present study. After 2 weeks of treatment, the mice were euthanized by cervical dislocation under deep isoflurane anesthesia (5% induction). Tumor tissues were then collected for histological examination, immunohistochemistry, immunofluorescence, and flow-cytometric analysis. All procedures were approved by the Institutional Animal Care and Use Committee of Shanghai Rat&Mouse Biotech Co., Ltd. (approval no. 20250514(12)) and were conducted in accordance with the Guide for the Care and Use of Laboratory Animals and ARRIVE guidelines.

### 2.17 Flow cytometry

Peripheral-blood mononuclear cells and tumor-infiltrating cells were isolated from mice in the vehicle-control and puerarin-treatment groups. Cell suspensions were washed with PBS containing 2% BSA and incubated for 30 min at 4 °C in the dark with anti-mouse/human CD11b-FITC (clone M1/70; BioLegend, San Diego, CA, USA; Cat. No. 101206) and anti-mouse F4/80-PE (clone BM8; BioLegend; Cat. No. 123110). After three washes with cold PBS, samples were acquired using a BD FACS LSR II flow cytometer and analyzed with FlowJo software. Following exclusion of debris and doublets, macrophage-lineage cells were quantified as F4/80^+^CD11b^+^ events. Only cell-surface staining was performed. No fixation/permeabilization, intracellular ALDH2 staining, or M1/M2 phenotyping panel was used; therefore, the flow-cytometry results quantify the abundance of F4/80^+^CD11b^+^ cells but do not establish macrophage-specific ALDH2 expression or functional polarization.

### 2.18 Statistical analysis

Data are presented as mean ± standard deviation (SD). Intergroup differences were evaluated using one-way analysis of variance or Student’s t-test using IBM SPSS Statistics (version 30.0; IBM Corp., Armonk, NY, USA), with statistical significance set at *p* < 0.05.

## 3 Results

### 3.1 Integrated multi-database analysis identified candidate therapeutic targets of puerarin for NSCLC

We obtained 25, 103, and 10 puerarin drug target genes from the Similarity Ensemble Approach, Swiss Target Prediction, and STITCH databases, respectively. A total of 130 drug targets were obtained after removing duplicates and taking the union. A total of 4,924 NSCLC-associated genes were retrieved from GeneCards, and 9,176 from CTD, resulting in 10,871 non-redundant disease-related genes. Differential expression analysis yielded 4,690 DEGs, among which 2,536 were upregulated and 2,154 were downregulated. Among the DEGs (adjusted *P*-value < 0.05), the top 10 ranked by |log_2_FC| were labeled on the volcano plot (**Figure 1A**) and visualized in the heatmap (**Figure 1B**). Candidate targets were identified from the intersection of 4,690 DEGs, 130 drug targets, and 10,871 genes associated with NSCLC (**Figure 1C**).

**Figure 1:**
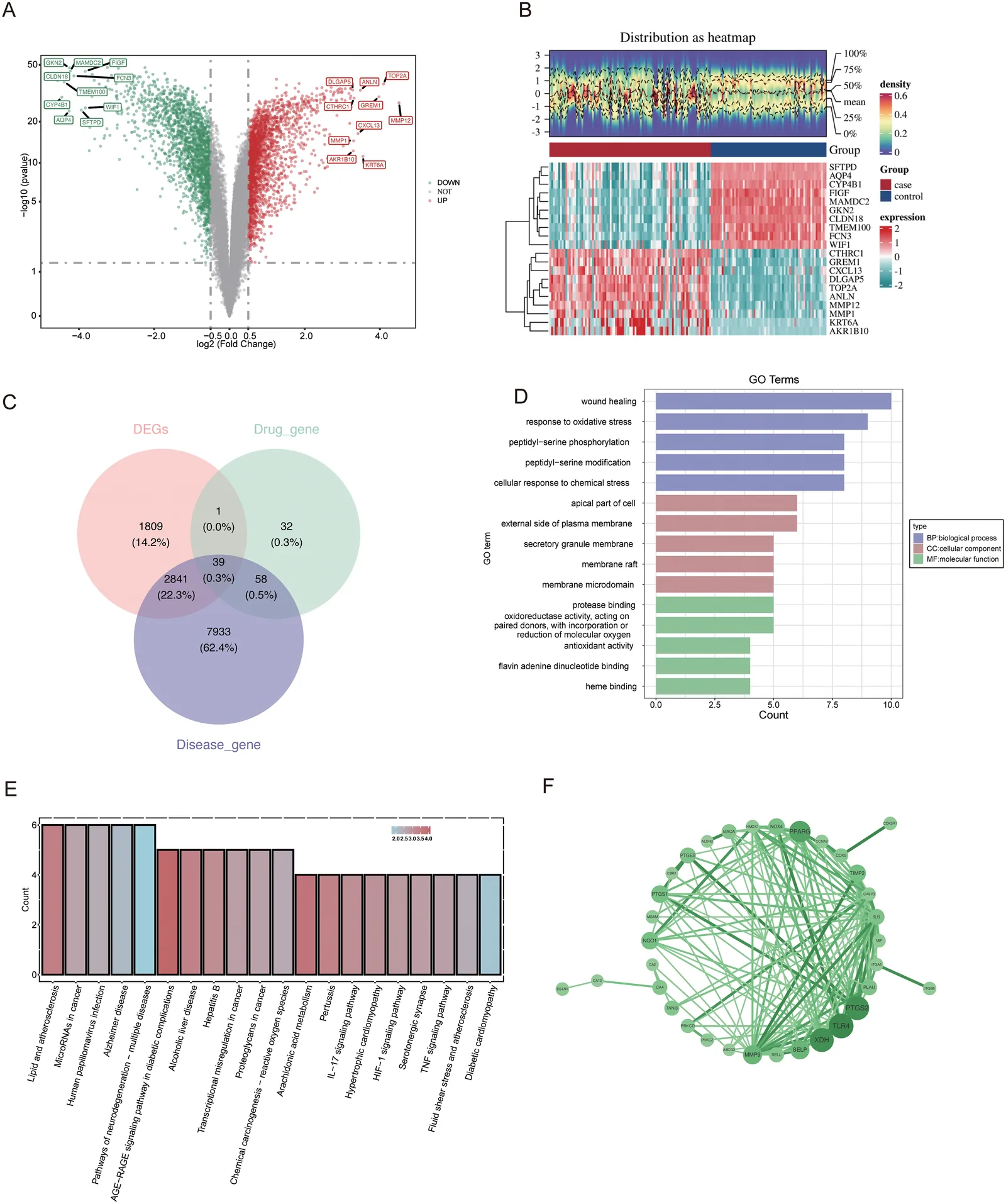
Screening and analysis of candidate genes. **(A)** Volcano plot of all identified differentially expressed genes (DEGs), with the top 10 genes (ranked by |log_2_FC|) labeled. **(B)** Heatmap displaying the expression profiles of the top 10 DEGs. **(C)** Venn diagram illustrating the intersections among 4,690 DEGs, 130 drug targets, and 10,871 non-small cell lung cancer (NSCLC)-related genes. **(D)** Gene Ontology (GO) enrichment analysis of the intersecting genes, highlighting the most relevant biological processes (BP), molecular functions (MF), and cellular components (CC). **(E)** Kyoto Encyclopedia of Genes and Genomes (KEGG) pathway enrichment analysis showing the top 20 significantly enriched pathways. **(F)** Protein-protein interaction (PPI) network of the intersecting genes, where nodes and edges represent proteins and their interactions, respectively.

GO enrichment analysis revealed 1,189 significant terms under the specified screening criteria. The analysis identified 1,011 biological process (BP) terms enriched in processes, including wound healing, response to oxidative stress, peptidyl-serine phosphorylation and modification, and cellular response to chemical stress; 114 molecular function (MF) terms enriched in functions, including protease binding, oxidoreductase and antioxidant activities, flavin adenine dinucleotide binding, and heme binding; 64 cellular component (CC) terms enriched in components, including the apical part of the cell, external side of the plasma membrane, secretory granule membrane, membrane raft, and membrane microdomain. The top five terms ranked by gene count for each category (BP, MF, CC) were visualized in **Figure 1D**. Furthermore, KEGG pathway analysis identified 50 significantly enriched pathways, primarily associated with Lipid and atherosclerosis, MicroRNAs in cancer, Human papillomavirus infection, Alzheimer’s disease, Pathways of neurodegeneration - multiple diseases, and the AGE-RAGE signaling pathway in diabetic complications. Pathways were prioritized by the gene count they comprised, with the top 20 selected for display (**Figure 1E**). Finally, the intersecting genes were used to construct a PPI network, which was visualized using Cytoscape software (**Figure 1F**).

### 3.2 Three machine learning models collectively identified five core prognostic genes for NSCLC

Three machine learning algorithms were employed to screen for potential prognostic genes from the 39 intersecting genes. LASSO logistic regression generated a selection path plot (**Figure 2A**) and coefficient profiles of the 39 variables (**Figure 2B**). At the optimal lambda.min value (~0.006), 14 feature genes were selected: MIF, SELP, ALDH2, ABCG2, PTGS1, MGAM, NQO1, NOX4, HMOX1, PDE4D, ITGA5, XDH, CBR1, and SQLE.

**Figure 2:**
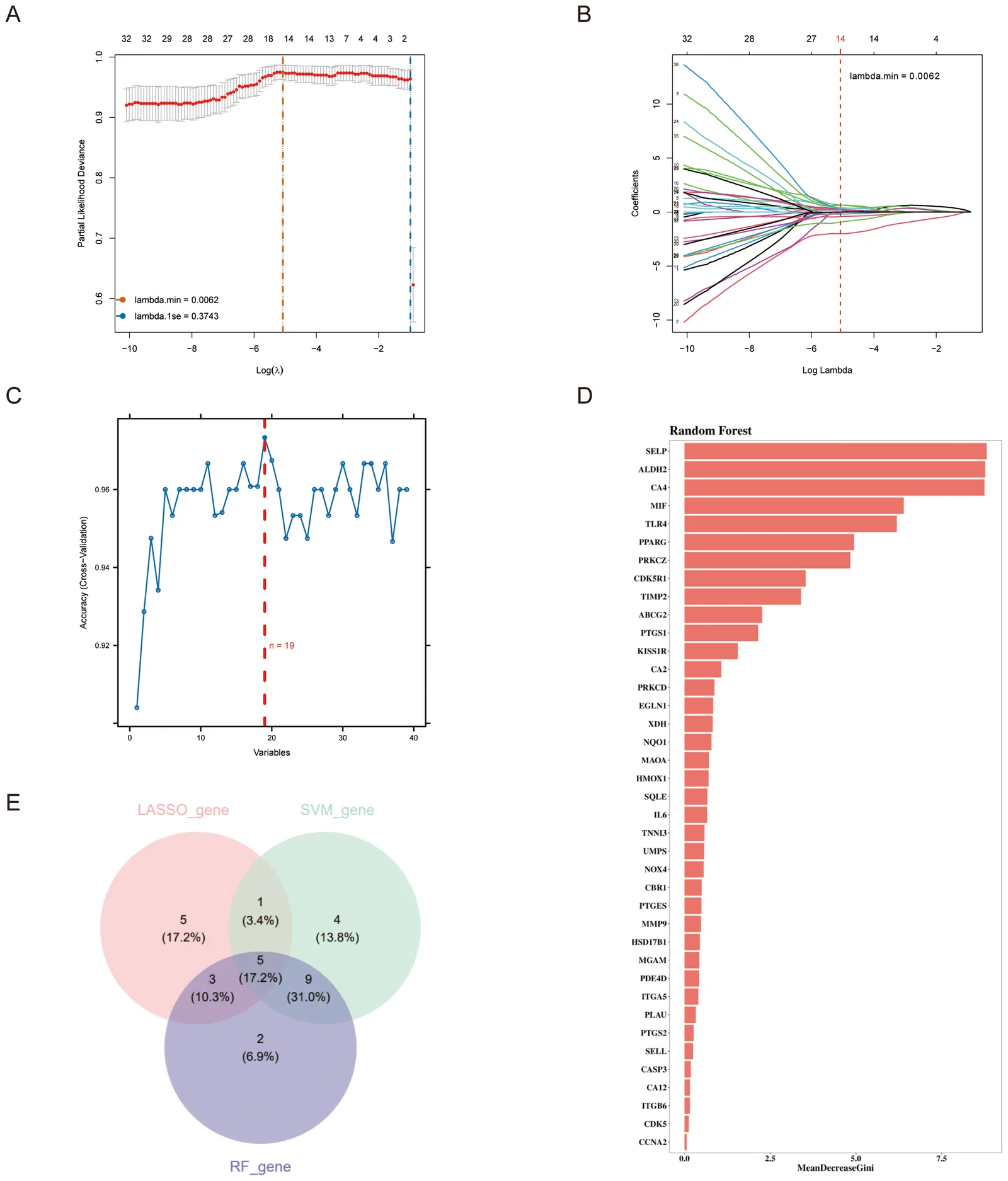
Comparison of feature selection results using Least Absolute Shrinkage and Selection Operator (LASSO), Support Vector Machine (SVM), and Random Forest (RF) algorithms. **(A)** Cross-validation curve for LASSO regression. Partial likelihood deviance is plotted against log(λ). Vertical dashed lines denote the optimal penalty parameters selected by the minimum criterion (λ.min = 0.0062) and the one‑standard‑error criterion (λ.lse = 0.3743). **(B)** Coefficient path plot for LASSO regression. Coefficient paths are shown against log(λ). As the penalty increases, coefficients of candidate genes shrink toward zero; the vertical line indicates the non‑zero coefficients retained at λ.min. **(C)** Feature selection process of SVM‑RFE. Cross‑validation accuracy is plotted against the number of variables, with the curve peaking at approximately 19 variables. **(D)** Ranking of feature importance based on RF. Genes are ranked by MeanDecreaseGini values (x‑axis); longer bars indicate greater contribution to classification accuracy. **(E)** Venn diagram showing the overlap of feature genes selected by the three models; the intersection identifies the final core prognostic genes.

Similarly, a SVM model was constructed using the same 39 intersecting genes. The SVM machine learning process identified 19 genes: MIF, CA4, SELP, ALDH2, PPARG, TLR4, PRKCZ, CDK5R1, CCNA2, ABCG2, EGLN1, KISS1R, PTGS1, TIMP2, MAOA, MGAM, IL6, MMP9, and HSD17B1 (**Figure 2C**).

RF model was also built based on these genes. Based on the criterion of importance value exceeding the median (mean decrease Gini), 19 candidate feature genes were retained: CA4, MIF, SELP, PPARG, ALDH2, TLR4, PRKCZ, ABCG2, PTGS1, EGLN1, TIMP2, CA2, KISS1R, MAOA, NQO1, PRKCD, CDK5R1, HMOX1, and XDH (**Figure 2D**).

By intersecting the results from the three approaches—14 genes from LASSO, 19 from SVM, and 19 from RF—five core prognostic genes were ultimately identified for subsequent analysis: ABCG2, ALDH2, MIF, PTGS1, and SELP (**Figure 2E**).

### 3.3 Four key targets (ABCG2, ALDH2, MIF, and SELP) form a highly accurate diagnostic model for NSCLC

ABCG2, ALDH2, MIF, and SELP were identified as the four key targets, exhibiting significant and consistent expression trends in both the training and validation cohorts (**Figures 3A, B**). Among them, MIF was upregulated, whereas ABCG2, ALDH2, and SELP were downregulated. ROC curves were generated for these genes in the training (**Figure 3C**) and validation (**Figure 3D**) sets. Genes with AUC values exceeding 0.7 in both datasets were considered key targets; as all four genes met this criterion, they were retained for subsequent analyses.

**Figure 3:**
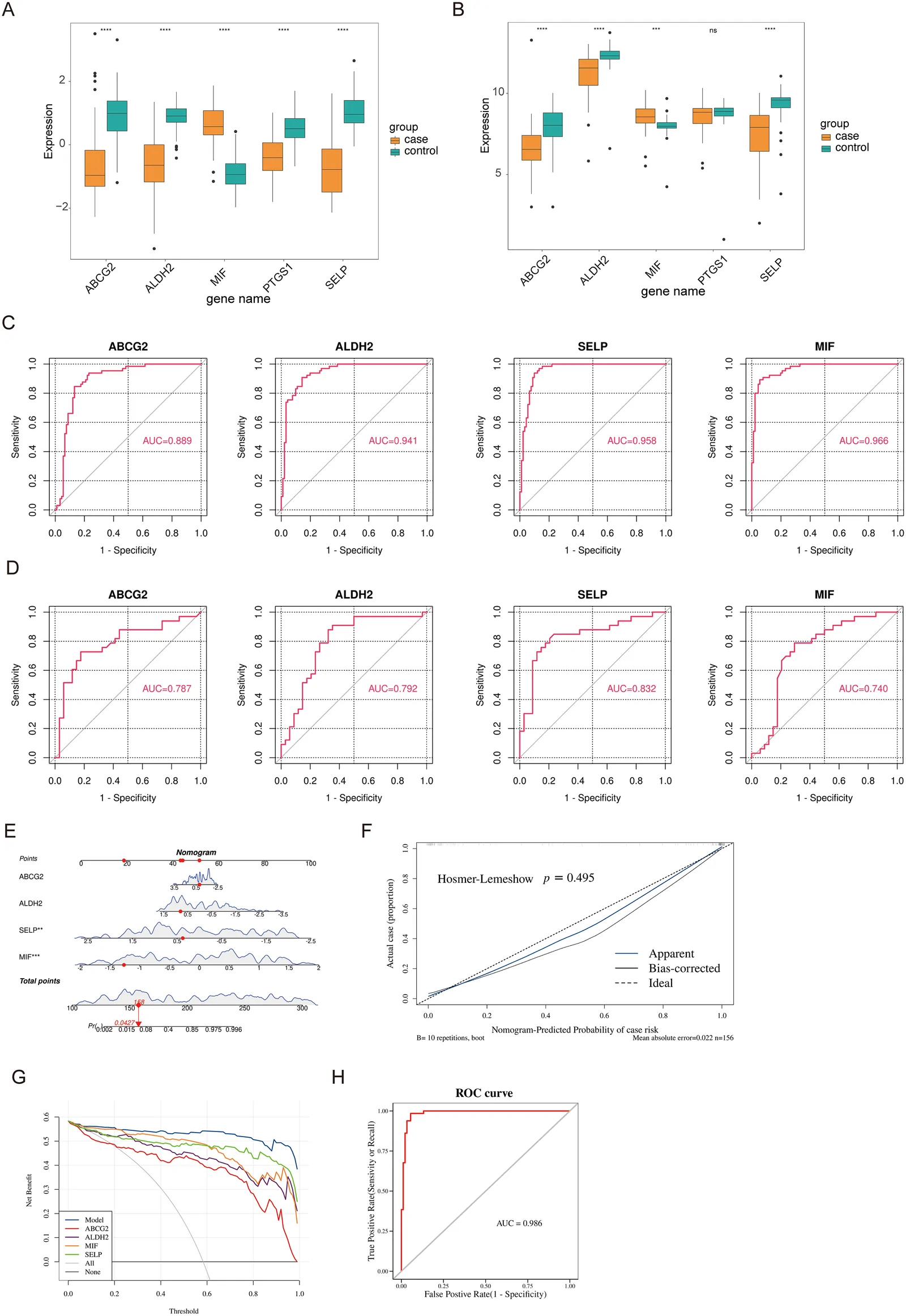
Identification and validation of a diagnostic nomogram for NSCLC. **(A, B)** Expression profiles of ABCG2, ALDH2, MIF, PTGS1, and SELP in the training **(A)** and validation **(B)**. **(C, D)** Receiver operating characteristic (ROC) curves for the four consistently differentially expressed genes (ABCG2, ALDH2, SELP, and MIF) in the training **(C)** and validation **(D)** sets. The area under the curve (AUC) values quantify the individual diagnostic performance of each biomarker. **(E)** Nomogram combining these four targets for individualized risk prediction. **(F)** Calibration curves showing the predictive accuracy of the nomogram (Ideal: reference line; Apparent: unadjusted performance; Bias-corrected: bootstrap-corrected performance; Hosmer-Lemeshow test *P* = 0.495). **(G)** Decision curve analysis (DCA) curve assessing the clinical utility of the nomogram. **(H)** ROC curve evaluating the comprehensive predictive capability of the nomogram model. Data are presented as means ± SEM, **P < 0.01, ***P < 0.001, ****P < 0.0001; ns, not significant.

A diagnostic nomogram incorporating these four targets was constructed using the training set to predict NSCLC risk (**Figure 3E**). The model's reliability was evaluated via a calibration curve, which yielded a *p*-value of 0.495 (>0.05), indicating good fit and high predictive accuracy (**Figure 3F**). DCA further confirmed the nomogram's clinical utility, demonstrating a superior net benefit compared to any single factor (**Figure 3G**). The ROC curve for the nomogram showed excellent discriminative ability, with an AUC of 0.986 (**Figure 3H**). To elucidate the potential biological functions of these key genes, GSEA was performed, and the top five most significantly enriched pathways were selected for presentation (**Supplementary Figure 1**). ABCG2 was significantly involved in cell cycle, complement and coagulation cascades, DNA replication, lysosome, and cytokine-cytokine receptor interaction (**Supplementary Figure 1A**). ABCG2 might serve as a mediator through which puerarin modulates these pathways in the context of NSCLC. ALDH2 played a significant role in complement and coagulation cascades, cell cycle, graft-versus-host disease, lysosome, and DNA replication (**Supplementary Figure 1B**). Furthermore, MIF was significantly associated with DNA replication, complement and coagulation cascades, cell cycle, cell adhesion molecules, and pyrimidine metabolism (**Supplementary Figure 1C**). Moreover, SELP was significantly associated with complement and coagulation cascades, cell cycle, graft-versus-host disease, hematopoietic cell lineage, and DNA replication (**Supplementary Figure 1D**).

### 3.4 Molecular docking and MD simulations of puerarin with ABCG2 and ALDH2

Molecular docking was performed to evaluate the binding affinities between the core active component puerarin and the key targets, and the resulting binding energies are listed in **Supplementary Table 1**. Binding energies < −5.0 kcal/mol and –7.0 kcal/mol were categorized as moderate and high affinity, respectively (25). Puerarin exhibited the highest affinity for ABCG2 (−9.2 kcal/mol), followed by ALDH2 (−9.0 kcal/mol), SELP (−7.6 kcal/mol), and MIF (−5.8 kcal/mol). The docking poses of puerarin with ABCG2, ALDH2, MIF, and SELP are shown in **Figures 4A–D**, respectively.

**Figure 4:**
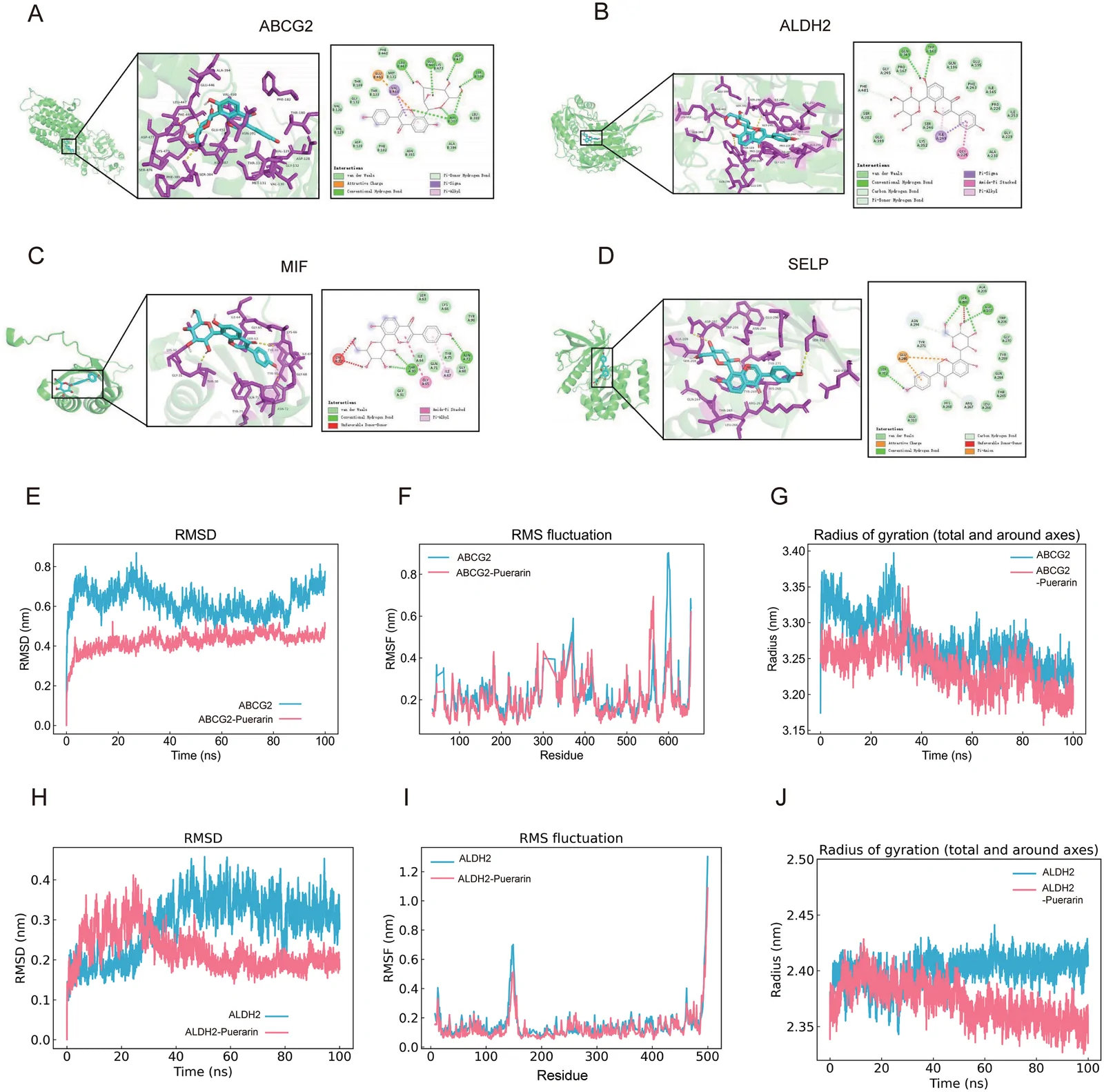
Molecular docking analysis of puerarin with four target proteins and molecular dynamics (MD) simulation of ABCG2 and ALDH2. **(A-D)** Molecular docking analyses showing the 3D binding modes (with enlarged views) and 2D interaction diagrams of puerarin with ABCG2 **(A)**, ALDH2 **(B)**, MIF **(C)**, and SELP **(D)**. **(E-G)** MD simulation results of ABCG2 and the ABCG2-puerarin complex over 100 ns: **(E)** Root mean square deviation (RMSD) of the backbone atoms; **(F)** Root mean square fluctuation (RMSF) of the residues; **(G)** Radius of gyration (Rg) of the total system. **(H-J)** MD simulation results of ALDH2 and the ALDH2-puerarin complex over 100 ns: **(H)** RMSD of the backbone atoms; **(I)** RMSF of the residues; **(J)** Rg of the total system.

Given their relatively high binding affinities, ABCG2 and ALDH2 were selected for MD simulations to assess the stability of the ligand–receptor interactions. The dynamic alterations within the monitored complex system were analyzed over a 100 ns simulation period, during which the root mean square deviation (RMSD), root mean square fluctuation (RMSF), and radius of gyration (Rg) were calculated.

RMSD was employed to monitor the conformational dynamics of the receptor-ligand complex, and the resulting curves reflect structural fluctuations. Furthermore, the RMSD trends for both the protein and ligand serve as critical indicators for assessing whether the system has reached equilibrium. As shown in **Figure 4E**, the RMSD of the ABCG2 protein alone reached a plateau after approximately 20–30 ns, fluctuating within 0.55–0.7 nm, whereas the ABCG2–puerarin complex stabilized earlier (within 10–20 ns) and exhibited lower RMSD values (0.4–0.5 nm). Similarly, the ALDH2 protein and its complex with puerarin both approached equilibrium after 40 ns of simulation, with the complex showing a decreased and subsequently stabilized RMSD trajectory (**Figure 4H**). These observations suggest that puerarin binding may correlate with enhanced conformational stability of both targets.

To characterize the residue-level dynamics underlying structural fluctuations in the complex system, the RMSF for each amino acid residue was calculated over the simulation trajectory. RMSF quantifies the time-averaged positional deviation of atoms, with higher values indicating greater local conformational flexibility. As presented in **Figures 4F** and **4I**, comparative RMSF profiles of the target protein and the protein–puerarin complex revealed that the majority of residues maintained relatively low flexibility. Notably, a slight reduction in residue flexibility was observed upon puerarin binding, suggesting a rigidification of the local structure and enhanced conformational stability.

Rg was employed to assess the compactness and folding tightness of the systems; lower Rg values typically indicate denser and more constrained conformations, whereas higher values reflect looser peptide chains (25). As shown in **Figures 4G** and **4J**, the Rg values of the target proteins alone were generally higher than those of the corresponding complexes, suggesting that puerarin binding may promote more compact structural packing.

The ABCG2–puerarin and ALDH2–puerarin models displayed relatively stable RMSD, RMSF, and Rg profiles during the simulations, suggesting favorable structural compatibility under the modeled conditions. However, these computational findings do not demonstrate direct physical binding or cellular target engagement and require confirmation by biochemical or biophysical assays.

### 3.5 MR analysis suggested an inverse association between genetically predicted ALDH2 expression and LUAD risk

Using the LUAD outcome dataset finn-b-C3_NSCLC_ADENO, the IVW analysis (**Supplementary Table 2**) suggested inverse associations for genetically predicted ALDH2 and MIF expression. No statistically significant heterogeneity or directional pleiotropy was detected in the available sensitivity analyses(**Supplementary Table 3**). Because ALDH2 was downregulated in both expression datasets and showed an odds ratio below 1, the direction of the genetic association was concordant with the observational expression pattern. In contrast, MIF was upregulated in NSCLC tissues but also showed an odds ratio below 1, producing a directionally discordant result. Given the limited number of LUAD cases in the outcome GWAS, these estimates should be interpreted cautiously as supportive genetic evidence rather than definitive causal effects.

To assess the correlation between exposure factor and outcome, a scatter plot (**Figure 5A**) was generated based on ALDH2 results. The x-axis illustrates the impact of SNPs on exposure, and the y-axis depicts their impact on the outcome. The colored lines represent the causal estimates derived from different MR algorithms. An intercept near zero indicates the absence of horizontal pleiotropy, suggesting minimal confounding influence and reinforcing the reliability of the findings. A non-zero intercept would imply the potential presence of directional pleiotropy. The results demonstrate a significant negative causal relationship between ALDH2 and the outcome, with consistent trends observed across all five algorithms.

**Figure 5:**
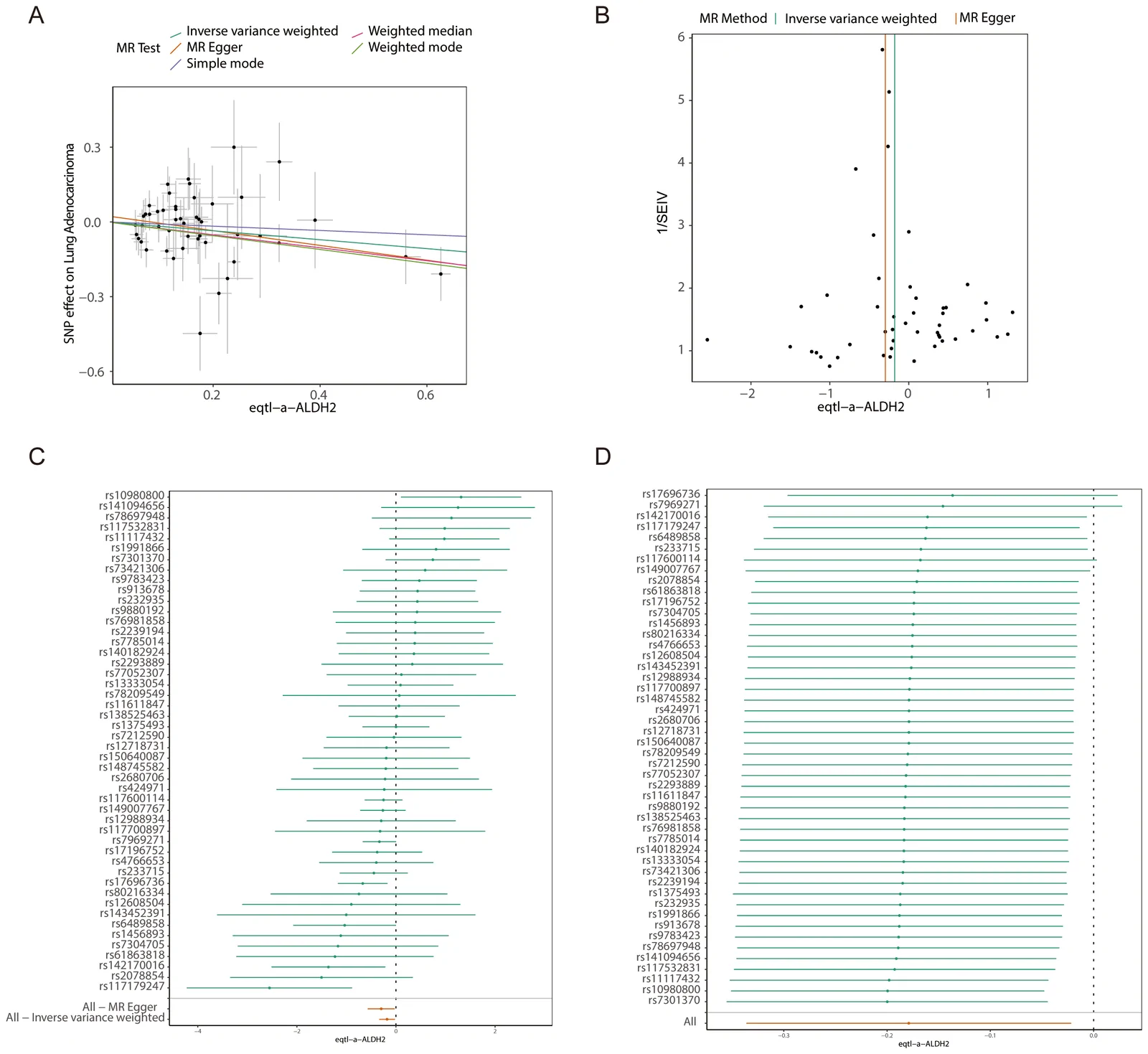
Mendelian randomization (MR) analysis of the causal effect of ALDH2 expression on Lung Adenocarcinoma (LUAD) risk. **(A)** Scatter plot illustrating the effect estimates of single nucleotide polymorphisms (SNPs) on ALDH2 expression (x-axis) versus their effects on LUAD risk (y-axis). The colored lines represent the causal estimates derived from five distinct MR methods (Inverse variance weighted (IVW), MR Egger, Weighted median, Simple mode, and Weighted mode), with intercepts near zero indicating minimal pleiotropy. **(B)** Funnel plot displaying the causal estimates (x-axis) against the inverse of standard errors (y-axis) to evaluate potential directional pleiotropy and publication bias. The vertical lines represent the combined estimates from the IVW and MR Egger methods. **(C)** Forest plot showing the causal effects of individual ALDH2-related SNPs on LUAD risk, alongside the pooled estimates calculated by MR Egger and Inverse variance weighted methods at the bottom. **(D)** Leave-one-out sensitivity analysis demonstrating the robustness of the causal association after sequentially excluding each individual SNP.

A funnel plot (**Figure 5B**) was drawn to evaluate potential heterogeneity and publication bias. The SNPs were distributed symmetrically, indicating the absence of significant directional pleiotropy and confirming the robustness of the estimates. A forest plot (**Figure 5C**) was constructed to evaluate the causal effect of individual SNP loci on the outcome. A value > 0 indicates that the specific SNP suggests an increase in exposure elevates disease risk; however, a value < 0 indicates that increasing exposure reduces disease risk. Under the IVW method, an increase in the ALDH2 exposure factor decreased disease risk, which aligns with previous results, indicating that the instrumental variables are either uncorrelated or only weakly correlated with the outcome. **Figure 5D** displays the leave-one-out analysis results of ALDH2 in relation to LUAD. Sequential removal of each SNP revealed a minimal impact on the outcome variable by the remaining SNPs, suggesting that the MR analysis results are reliable and stable.

### 3.6 Mapping the cellular sources and immune correlates of four key genes via single-cell and bulk RNA-seq

For the training set, a heatmap illustrating the enrichment scores of 28 immune cell infiltrates across the two sample groups was generated (**Figure 6A**). The results revealed that activated CD4^+^ T cells exhibited the highest infiltration score in the disease group. In contrast, effector memory CD8^+^ T cells, MDSC, neutrophils, eosinophils, and mast cells demonstrated higher infiltration scores in the control group. A total of 24 significantly distinct infiltrating immune cells were identified between the two groups (**Figure 6B**). Subsequently, correlation analysis among these differential immune cells was performed and visualized in a heatmap (**Figure 6C**). The strongest significant negative correlation was observed between activated/memory B cells and immature dendritic cells (cor = –0.33, *p* < 0.001), whereas the strongest significant positive correlation was observed between MDSC and effector memory CD8^+^ T cells (cor = 0.89, *p* < 0.001). An analysis was conducted on the correlations between the four main target genes and the 24 distinct immune cells (**Figure 6D**). The results revealed significant correlations between genes and immune cells: ABCG2 with mast cells (cor = 0.63, *p* < 0.001), ALDH2 with immature dendritic cells (cor = 0.71, *p* < 0.001), SELP with mast cells (cor = 0.84, *p* < 0.001), and MIF with eosinophils (cor = –0.73, *p* < 0.001).

**Figure 6:**
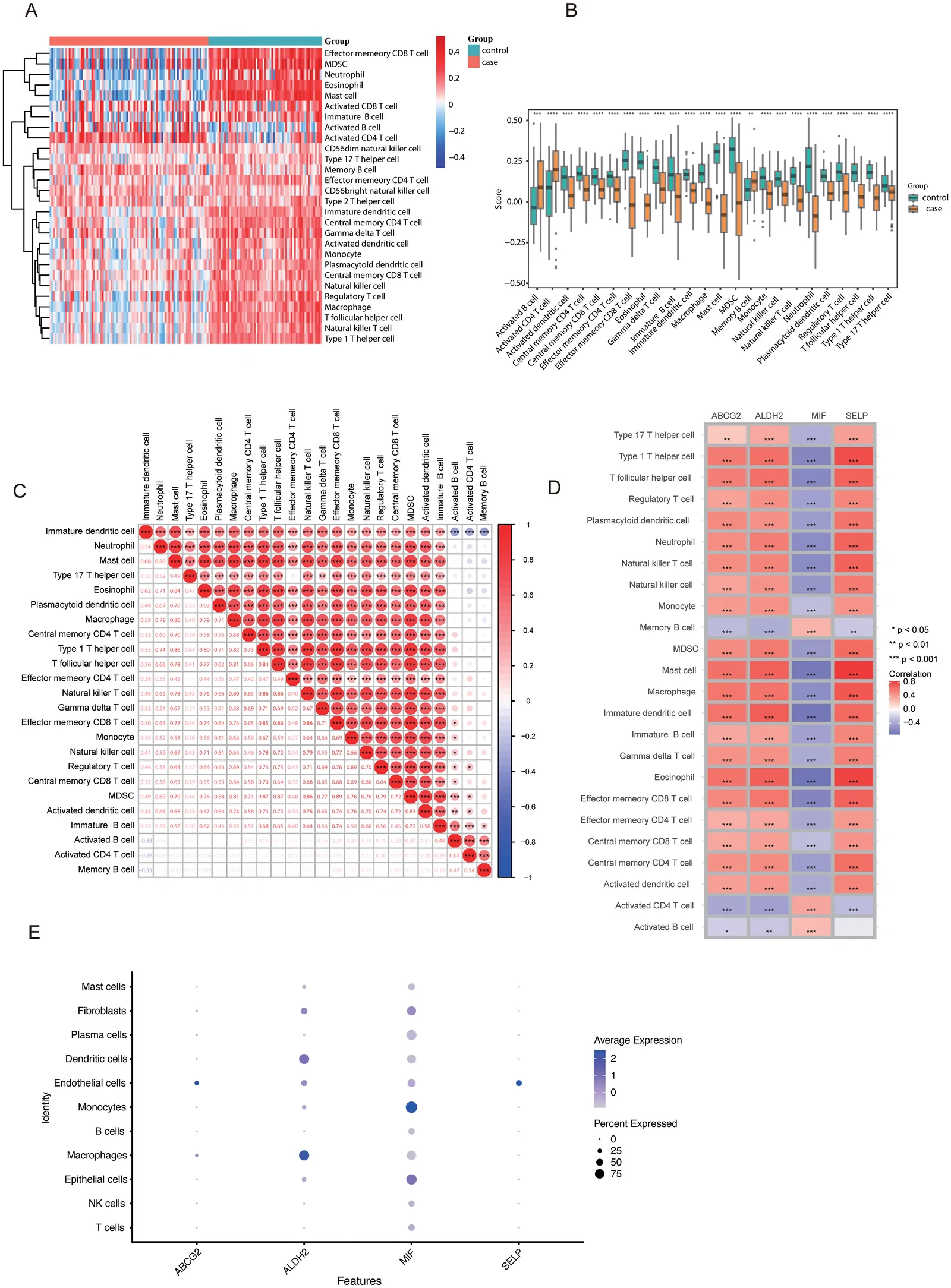
Analysis of immune infiltration and expression of four key target genes based on bulk and single-cell RNA sequencing data. **(A–D)** Bulk transcriptome sequencing analysis. **(A)** Heatmap depicting the enrichment scores of 28 immune cell types between the disease (Case) and control groups. The color scale ranges from blue (low enrichment) to red (high enrichment). **(B)** Box plot showing the significantly differential immune cells between the Case and Control groups. Asterisks indicate statistical significance. **(C)** Correlation matrix of the differential immune cells. The size of the circles and the color intensity (red to blue, red representing positive, blue representing negative) indicate the strength and statistical significance of the correlations. **(D)** Correlation heatmap between four key target genes (ABCG2, ALDH2, MIF, SELP) and 24 differential immune cells. Red and blue denote positive and negative correlations, respectively. Asterisks indicate statistical significance. **(E)** Bubble plot of the expression of the four key target genes across 11 major cell types based on single-cell RNA sequencing. The x-axis indicates the genes, and the y-axis indicates the cell types. The dot size represents the percentage of cells expressing the gene, while the color intensity indicates the average expression level. *P < 0.05, **P < 0.01, ***P < 0.001, ****P < 0.0001.

Single-cell analysis provided bubble charts revealing the expression of key targets across various cells. A total of 151,076 cells and 25,370 genes were initially obtained; after data filtering, 137,725 cells remained, with the gene count unchanged. To exclude low-quality cells, we evaluated the mitochondrial gene ratio, an indicator of cell damage, with a higher ratio indicating lower cell viability. Quality control results before and after processing are presented in **Supplementary Figures 2A, B**, respectively. The top 2,000 highly variable genes were selected for downstream analysis (**Supplementary Figure 2C**) to reduce computational complexity. PCA was then applied to the data from different samples. **Supplementary Figure 2D** demonstrates that cells from control and disease groups were co-clustered, suggesting the absence of significant batch effects.

Different colors indicate various clusters, resulting in the division of all cells into 24 clusters, as illustrated in **Supplementary Figure 2E**. Subsequently, we identified the cell types for each cluster. The 24 clusters were manually annotated and categorized into 11 distinct cell subsets: T cells, natural killer cells, monocytes, endothelial cells, epithelial cells, macrophages, B cells, fibroblasts, mast cells, plasma cells, and dendritic cells. The distribution of these 11 cell subsets is depicted in **Supplementary Figure 2F**, with the separation of normal and tumor groups shown side by side in **Supplementary Figure 2G**. Based on the marker genes used for cluster identification (**Supplementary Table 4**), their expression levels are visualized in **Supplementary Figure 2H**. We then examined the expression patterns of key target genes across different cell types. As shown in the bubble plot (**Figure 6E**) ABCG2 was predominantly expressed in endothelial cells, ALDH2 was enriched in macrophages, MIF was highly expressed in monocytes, and SELP was also highly expressed in endothelial cells.

### 3.7 *In vivo* puerarin treatment inhibited LUAD growth and was accompanied by ALDH2 upregulation and increased macrophage infiltration

Based on the integrative prioritization described above, ALDH2 was selected for tissue-level evaluation. TCGA analysis showed lower ALDH2 expression in LUAD and LUSC tissues than in corresponding non-cancerous tissues (**Figure 7A**), and higher ALDH2 expression was associated with better overall survival in LUAD (**Figure 7B**). In the orthotopic LLC-luc model, puerarin treatment reduced tumor burden, decreased Ki67 staining, and increased total ALDH2 immunohistochemical signal (**Figures 7C–F**). Immunofluorescence showed increased ALDH2 and F4/80 signals within overlapping tumor regions after treatment(**Figure 7G**). This spatial co-occurrence does not demonstrate that the increase in ALDH2 occurred specifically within macrophages. Flow cytometry further showed an increased proportion of F4/80^+^CD11b^+^ cells in peripheral blood and tumor tissue (**Figures 7H, I**). Because intracellular ALDH2 staining and M1/M2 or functional phenotyping were not performed, these results support an association among puerarin treatment, total ALDH2 upregulation, and increased macrophage abundance, but do not establish direct puerarin–ALDH2 binding, macrophage-specific ALDH2 induction, or macrophage reprogramming.

**Figure 7:**
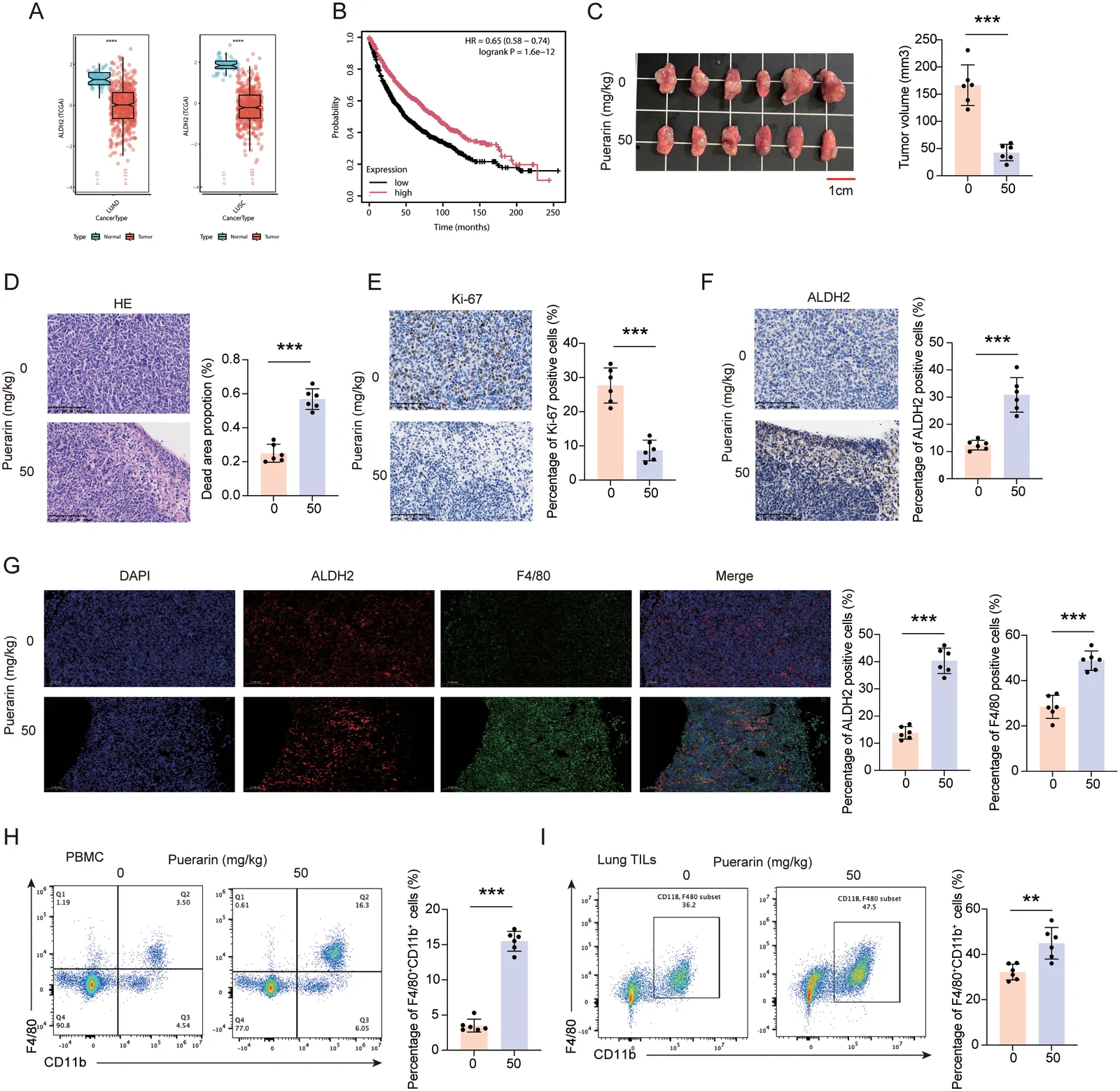
Tissue-level and *in vivo* evaluation of ALDH2 and macrophage abundance following puerarin treatment. **(A)** ALDH2 expression in LUAD and LUSC tissues and corresponding non-cancerous tissues. **(B)** Kaplan–Meier analysis of overall survival according to ALDH2 expression in LUAD. **(C)** Orthotopic tumor burden after vehicle or puerarin treatment. **(D)** Hematoxylin and Eosin staining. **(E)** Ki67 immunohistochemistry. **(F)** Total ALDH2 immunohistochemical staining. **(G)** Immunofluorescence images showing ALDH2 and F4/80 signals in tumor sections. Signal overlap indicates spatial co-occurrence at the tissue level and does not establish macrophage-specific ALDH2 upregulation. Scale bars, 100 μm. **(H)** Proportion of F4/80^+^CD11b^+^ cells in peripheral blood. **(I)** Proportion of F4/80^+^CD11b^+^ cells in tumor tissue. Only surface staining was performed; M1/M2 phenotyping and intracellular ALDH2 quantification were not conducted. Data are presented as means ± SEM, **P < 0.01, ***P < 0.001, ****P < 0.0001.

## 4 Discussion

Puerarin has reported anti-inflammatory, immunomodulatory, and anti-tumor activities (6, 13, 26–28), however, its candidate molecular targets and immune associations in lung cancer remain incompletely defined. Preliminary studies indicate potential anti-cancer activity. For example, puerarin has been shown to reduce A549 cell migration and suppress invasion-related factors by inhibiting extracellular signal-regulated kinase (ERK) phosphorylation (29), hinder NSCLC progression *in vitro* and *in vivo* partly via the miR-342/CCND1 axis (30), and notably suppress NSCLC proliferation while enhancing apoptosis (31). The specific molecular mechanism by which puerarin exhibits anti-cancer effects in NSCLC and its impact on the immune microenvironment of NSCLC remain unclear.

Network pharmacology is useful for organizing drug–target–disease relationships and generating hypotheses for multi-component or multi-target agents (32). Nevertheless, its outputs depend on database coverage, target-annotation quality, prediction algorithms, and the thresholds used for inclusion. These approaches generally do not incorporate drug exposure, tissue distribution, dose, metabolic transformation, cell state, or temporal context (33). Machine-learning convergence and multi-omics triangulation can reduce dependence on any single database, but they do not convert computational associations into definitive mechanisms. Therefore, the present computational results should be viewed as a prioritization framework that guided downstream evaluation rather than proof of a puerarin–ALDH2 pathway.

Among the four key targets, ALDH2 was prioritized for downstream evaluation based on convergence across multiple, independent criteria rather than any single result. It was consistently downregulated in the two bulk datasets, achieved diagnostic discrimination in both cohorts, showed favorable modeled interaction with puerarin, displayed a directionally concordant inverse MR association with LUAD risk, and was relatively enriched in macrophages in the exploratory single-cell dataset, and established biological relevance to aldehyde detoxification and redox homeostasis. In comparison, ABCG2 and SELP were mainly enriched in endothelial cells, while the direction of the MIF expression change was inconsistent with its MR estimate. This rationale explains the focus on ALDH2 but does not exclude ABCG2, MIF, SELP, or other molecules from contributing to puerarin’s likely multi-target effects.

Docking and MD simulations predicted favorable structural compatibility of puerarin with ABCG2 and ALDH2. Stable RMSD, RMSF, and Rg profiles indicate that the modeled complexes remain conformationally stable under the selected simulation parameters. However, such analyses cannot determine whether puerarin binds ALDH2 in cells, whether the predicted binding site is accessible in the native protein, or whether binding changes ALDH2 enzymatic activity. Direct interaction would require orthogonal biochemical or biophysical assays, and target dependence would require pharmacological inhibition, genetic knockdown or knockout, and rescue experiments. Accordingly, the structural analyses provide a rationale for experimental testing rather than confirmation of direct binding.

The MR analysis suggested that genetically predicted higher ALDH2 expression was associated with lower LUAD risk. This result is biologically compatible with the reduced ALDH2 expression observed in LUAD and with studies linking ALDH2 loss to aldehyde accumulation, DNA damage, and poorer outcomes (34, 35). Nevertheless, the outcome dataset contained only 571 cases, which substantially limits statistical power and can produce imprecise or unstable estimates despite nominally negative heterogeneity and pleiotropy tests. In addition, the whole-blood eQTL instruments may not fully represent ALDH2 regulation in lung tissue or tumor-associated macrophages. Both exposure and outcome datasets were predominantly European, limiting generalizability to East Asian and other populations in which ALDH2 functional variants and allele frequencies differ. The MR result should therefore be considered supportive evidence for prioritization and not definitive proof of a causal protective effect across all patients with NSCLC.

ALDH2, a member of the acetaldehyde dehydrogenase family, comprises 517 amino acids and four identical subunits that form a stable homotetramer. It plays a critical role in redox reactions involving ethanol and aldehydic products from lipid peroxidation (36). Its primary function is to convert acetaldehyde (ACE) into nontoxic acetic acid. A well-established association exists between ALDH2 dysfunction and the development, progression, and metastasis of various tumor types (37, 38). In LUAD, ALDH2 deficiency also exerts a significant effect on tumor development and progression. Multiple studies have shown that ALDH2 expression is markedly downregulated at both the mRNA and protein levels in LUAD patient samples. Furthermore, the AJCC has reported a significant correlation between reduced ALDH2 expression and decreased overall survival across different stages of LUAD (39). Another study found that ALDH2 suppression correlates with poor prognosis in LUAD, attributed to ACE accumulation and enhanced DNA damage (40).

ALDH2 also plays a vital role in regulating immune cell function within the tumor microenvironment, a focal point of ongoing research in cancer biology (35). A comprehensive cancer study identified a strong positive correlation between ALDH2 expression and the infiltration of CD4^+^ T cells, CD8^+^ T cells, neutrophils, B cells, and macrophages, across various tumor types including LUAD (41, 42).

ALDH2 disrupts the reactive oxygen species (ROS)/Nrf2 pathway to enhance autophagy, thus inhibiting immune evasion in liver hepatocellular carcinoma (43). ALDH2 protects against acute kidney injury by promoting autophagy activation and eliminating ROS via the Beclin-1 pathway (44). Overexpression of ALDH2 has been shown to inhibit ROS production and the NF-κB/vascular endothelial growth factor C (VEGFC) oncogenic pathway (45). A plausible hypothesis is that increased ALDH2 activity could reduce aldehyde adduct formation and mitochondrial ROS, preserving metabolic fitness and altering redox-sensitive pathways such as Nrf2, NF-κB, and autophagy. Tumor-associated macrophages are crucial components of the immunosuppressive microenvironment. Macrophage function and polarization are closely linked to altered metabolism. Because inflammatory macrophage states are commonly associated with enhanced glycolytic and reactive oxygen/nitrogen metabolism, whereas reparative macrophage states rely more strongly on mitochondrial oxidative metabolism (46), ALDH2-dependent control of aldehyde stress could influence macrophage activation thresholds, cytokine production, or survival. Mechanistically, ALDH2 deficiency augments atherosclerosis through the ubiquitin-specific protease 14 (USP14)-cyclic GMP-AMP synthase (cGAS)-stimulator of interferon genes (STING)-dependent polarization of proinflammatory macrophages (47). In bladder cancer, baicalin exerts potent anti-tumor effects by upregulating ALDH2, thereby suppressing programmed death-ligand 1 (PD-L1)-mediated M2 macrophage polarization and remodeling the tumor immune microenvironment (48). Although these pathways are mechanistically plausible, they were not assessed in the current study and should be regarded as hypotheses for future investigation rather than conclusions supported by the present data.

The immune findings also require cautious interpretation. Increased F4/80^+^CD11b^+^ abundance does not by itself indicate an anti-tumor macrophage phenotype, because tumor-associated macrophages are heterogeneous and may support or suppress tumor growth depending on their activation state, location, and metabolic program. The immunofluorescence images demonstrate spatial overlap of ALDH2 and F4/80 signals at the tissue level but cannot determine whether ALDH2 expression increased within individual macrophages. Likewise, the flow-cytometry panel quantified total macrophage-lineage cells but did not include CD86, MHC-II, iNOS, CD206, Arg1, intracellular cytokines, phagocytosis, or intracellular ALDH2. The observed coexistence of tumor suppression, increased total ALDH2, and increased macrophage abundance is therefore an association that motivates mechanistic experiments rather than evidence that puerarin converted macrophages into a defined anti-tumor state.

Several limitations should be emphasized. First, direct puerarin–ALDH2 target engagement and ALDH2-dependent anti-tumor activity were not tested using biochemical binding assays, ALDH2 inhibitors, siRNA, knockout models, or rescue experiments. Second, macrophage-specific ALDH2 expression and functional polarization were not measured, and the flow-cytometry analysis was limited to surface F4/80 and CD11b. Third, the single-cell dataset included paired samples from only two patients, making it suitable for exploratory cell mapping but not for population-level inference or assessment of interpatient heterogeneity. Fourth, the MR outcome had a small case number and was restricted to European ancestry; the exposure instruments were derived from whole blood rather than lung or tumor macrophages. Fifth, the animal validation used one murine model, one puerarin dose, and a limited sample size, precluding assessment of dose–response, optimal dosing, pharmacokinetics, or model-specific effects. Sixth, no human LUAD cell line, primary human macrophage, patient-derived organoid, or human macrophage–tumor co-culture was used for mechanistic validation. Future studies should combine direct binding assays, macrophage-specific ALDH2 perturbation, multicolor and intracellular flow cytometry, functional phagocytosis and cytokine assays, metabolic flux and aldehyde/ROS measurements, dose-ranging studies, and validation in larger human cohorts.

## 5 Conclusion

In summary, this multi-omics and *in vivo* study highlights ALDH2 as a candidate puerarin-associated molecule in LUAD. Puerarin treatment not only inhibited orthotopic tumor growth but also elevated total ALDH2 expression and F4/80⁺CD11b⁺ macrophage abundance. Integrative transcriptomic, computational, genetic, single-cell, and animal data collectively suggest a possible interplay among puerarin, ALDH2, and the macrophage-rich tumor microenvironment. Nevertheless, direct molecular binding, macrophage-specific ALDH2 regulation, and the net functional impact of macrophage changes have not been proven. Thus, our results offer a hypothesis-generating framework for subsequent mechanistic and translational work, not a confirmed causative pathway.

## Data Availability

The original contributions presented in the study are included in the article/**Supplementary Material**. Further inquiries can be directed to the corresponding authors.
