# Supplementary material for: Integrative multi-omics analysis prioritizes ALDH2 as a candidate Puerarin-associated target linked to macrophage infiltration in lung adenocarcinoma
Source: Front Immunol. 2026 Sep 16;17:1918481. doi: 10.3389/fimmu.2026.1918481 (PMC13623940; doi:10.3389/fimmu.2026.1918481)
Supplement: Supplementary file 1 [file DataSheet1.doc]

Integrative multi-omics analysis prioritizes ALDH2 as a candidate Puerarin-associated target linked to macrophage infiltration in lung adenocarcinoma

Yufeng Chen^1†^, Zhujuan Zhong^1†^, Guoyuan Ju^2^, Yuanyuan Cai^1^, Lina Zhou^1^, Qianyu Wang^1*^ and Bo Yang^3*^

^1^Department of Pathology, Jiangsu Province (Suqian) Hospital, Suqian, Jiangsu, China, ^2^The Quzhou Affiliated Hospital of Wenzhou Medical University, Quzhou People’s Hospital, Quzhou, Zhejiang, China, ^3^Department of Oncology, The Affiliated Suqian Hospital of Nanjing University of Chinese Medicine，Suqian Hospital of Traditional Chinese Medicine，Suqian, Jiangsu, China

*Correspondence: Qianyu Wang: [13852423022@139.com](mailto:13852423022@139.com); Bo Yang: zyyyb188@163.com

^†^ Yufeng Chen and Zhujuan Zhong contributed equally to this work

**Supplementary materials:**

Supplementary Figure 1. GSEA enrichment results for the four key targets. Gene set enrichment analysis (GSEA) was performed to identify signaling pathways and biological processes associated with **(A)** ABCG2, **(B)** ALDH2, **(C)** MIF and **(D)** SELP.

**Supplementary Figure 2. Quality control, dimensionality reduction, and clustering analysis of single-cell RNA sequencing (scRNA-seq) data.**

**(A)** Violin plots displaying the distributions of quality control (QC) metrics—including nFeature_RNA, nCount_RNA, and percent.mt prior to QC filtering. nFeature_RNA represents the number of detected genes per cell, nCount_RNA represents the total UMI (transcript) counts per cell, and percent.mt indicates the proportion of mitochondrial reads per cell. **(B)** Violin plots illustrating the same QC metrics across samples after rigorous quality control filtering, demonstrating the removal of low-quality cells and doublets. **(C)** Visualization of highly variable genes selected for downstream dimensionality reduction and clustering analyses. **(D)** Principal component analysis (PCA) scatter plot showing the distribution and variance of all sampled cells across the top principal components, colored by sample origin. **(E)** UMAP (Uniform Manifold Approximation and Projection) plot revealing the global clustering architecture of all qualified cells, with each dot representing an individual cell. **(F)** UMAP plot depicting the identified cell subpopulations following graph-based clustering, with distinct colors assigned to each transcriptionally distinct cell subtype. **(G)** UMAP visualization of cellular clusters stratified by group. Clustering results for the Normal group and Tumor group are presented from left to right, respectively. **(H)** Expression profiles of representative marker genes across the identified cell clusters, highlighting the molecular signatures used for the annotation of each cell type or cluster.

**Supplementary Table 1**

Docking binding energies of active ingredients and key targets

| Gene | PDB | Drug | Docking score(kcal/mol) |
| --- | --- | --- | --- |
| ABCG2 | 6VXI | puerarin | -9.2 |
| ALDH2 | 8DR9 | puerarin | -9 |
| MIF | 4GUM | puerarin | -5.8 |
| SELP | 4GXB | puerarin | -7.6 |

**Supplementary Table 2**

Analysis results of MR randomization

| gene | outcome | method | nsnp | pval | or |
| --- | --- | --- | --- | --- | --- |
| ALDH2 | finn-b-C3_NSCLC_ADENO | Inverse variance weighted | 48 | 0.02561 | 0.83608 |
| MIF | finn-b-C3_NSCLC_ADENO | Inverse variance weighted | 10 | 0.01262 | 0.62906 |

**Supplementary Table 3**

Sensitivity analyses

| gene | het_qvalue | pleio_P |
| --- | --- | --- |
| ALDH2 | 0.12858 | 0.30331 |
| MIF | 0.91023 | 0.74581 |

**Supplementary Table 4**

Marker genes of cell cluster

| Cell_name | Marker_gene |
| --- | --- |
| NK cells | NKG7，GNLY，KLRD1 |
| T cells | CD2，CD3D，CD3E，CD3G |
| B cells | MS4A1，CD19，CD79B |
| Epithelial cells | EPCAM，KRT18，KRT8，CDH1 |
| Monocytes | S100A9，S100A8 |
| Fibroblasts | LUM，COL1A1，COL3A1，THY1，DCN，COL6A3 |
| Endothelial cells | CLDN5，PECAM1，VWF，TM4SF1 |
| Dendritic cells | LGALS2，CD86，PLD4，LILRA4 |
| Plasma cells | MZB1 |
| Mast cells | CTSG，ADCYAP1，TPSD1，GCSAML |
| Macrophages | MS4A7，CD68，CD163 |
